# *N*-Acetylaspartylglutamate (NAAG) Pretreatment Reduces Hypoxic-Ischemic Brain Damage and Oxidative Stress in Neonatal Rats

**DOI:** 10.3390/antiox9090877

**Published:** 2020-09-17

**Authors:** Ewelina Bratek, Apolonia Ziembowicz, Elzbieta Salinska

**Affiliations:** Department of Neurochemistry, Mossakowski Medical Research Centre, Polish Academy of Sciences, 02-106 Warsaw, Poland; aziembowicz@imdik.pan.pl (A.Z.); esalinska@imdik.pan.pl (E.S.)

**Keywords:** neonatal hypoxia-ischemia, *N*-acetylaspartylglutamate (NAAG), preconditioning, oxidative stress, TGF-β

## Abstract

*N*-acetylaspartylglutamate (NAAG), the most abundant peptide transmitter in the mammalian nervous system, activates mGluR3 at presynaptic sites, inhibiting the release of glutamate, and acts on mGluR3 on astrocytes, stimulating the release of neuroprotective growth factors (TGF-β). NAAG can also affect *N*-methyl-d-aspartate (NMDA) receptors in both synaptic and extrasynaptic regions. NAAG reduces neurodegeneration in a neonatal rat model of hypoxia-ischemia (HI), although the exact mechanism is not fully recognized. In the present study, the effect of NAAG application 24 or 1 h before experimental birth asphyxia on oxidative stress markers and the potential mechanisms of neuroprotection on 7-day old rats was investigated. The intraperitoneal application of NAAG at either time point before HI significantly reduced the weight deficit of the ischemic brain hemisphere, radical oxygen species (ROS) content and activity of antioxidant enzymes, and increased the concentration of reduced glutathione (GSH). No additional increase in the TGF-β concentration was observed after NAAG application. The fast metabolism of NAAG and the decrease in TGF-β concentration that resulted from NAAG pretreatment, performed up to 24 h before HI, excluded the involvement mGluR3 in neuroprotection. The observed effect may be explained by the activation of NMDA receptors induced by NAAG pretreatment 24 h before HI. Inhibition of the NAAG effect by memantine supports this conclusion. NAAG preconditioning 1 h before HI results in a mixture of mGluR3 and NMDA receptor activation. Preconditioning with NAAG induces the antioxidative defense system triggered by mild excitotoxicity in neurons. Moreover, this response to NAAG pretreatment is consistent with the commonly accepted mechanism of preconditioning. However, this theory requires further investigation.

## 1. Introduction

Perinatal hypoxia is the most common cause of death and damage to the central nervous system in fetuses and newborns. Deprivation of oxygen accompanied by a lack of perfusion to various organs, especially the brain, often leads to symptoms of abnormal neurological function termed neonatal hypoxic-ischemic encephalopathy (neonatal HIE). Currently, 2–4 out of every 1000 live newborns are diagnosed with neonatal HIE, and the incidence of neonatal HIE is particularly high in developing countries [1,2,3,4]. The mortality rate of hypoxia-ischemia (HI) in live newborns in these countries is 15%–20% [5]. Conditions such as premature birth enhance vulnerability to HI insults, increasing the incidence of motor and sensory deficits, cognitive impairments and emotional disorders [6,7]. The developing brain has a high metabolic need, and this makes them particularly vulnerable to HI. Interruption of blood and oxygen supplies during HI results in the loss of adenosine triphosphate (ATP), which reflects a disruption in the balance of ATP-dependent ions.

This results in excessive release of neurotransmitters, including glutamate, which activates α-amino-3-hydroxy-5-methyl-4-isoxazole-propionate (AMPA) and *N*-methyl-d-aspartate (NMDA) receptors, leading to excitotoxicity, which is characterized by intracellular Ca^2+^ accumulation, the activation of nitric oxide synthase, the impairment of mitochondrial function, and production of free radicals [8,9,10]. The induction of oxidative stress may initiate damage to proteins, lipids and DNA, leading to the initiation of programmed cell death [11,12]. Oxidative stress leads to increased production of radical oxygen species (ROS) and reactive nitrogen species (RNS), which function as signaling molecules under physiological conditions; however, increases in the concentration of these molecules in the hypoxic-ischemic brains make them extremely dangerous [13]. Additionally, the newborn brain is rich in iron ions, which play an important role in growth and development; however, unbound free iron ions also contribute to ROS formation through the Fenton reaction [14].

The developing brain is well equipped with antioxidant tools. Previous studies have shown that the activity of the main antioxidant enzymes, such as catalase, superoxide dismutase (SOD) and glutathione peroxidase (GPx), is increased by two- or three-fold in the immature brain and that the glutathione (GSH) concentration is significantly higher [15,16].

Interestingly, GSH not only plays an important role in intracellular antioxidant metabolic processes but is also involved in the eventual removal of detoxified oxidation products from the cells [17].

However, it seems that, under HI conditions, antioxidant enzymes cannot efficiently reduce the production of ROS, which results in the disruption of the physiological balance between ROS formation/neutralization and thus leading to neurodegeneration.

The current therapeutic strategies for neonatal oxidative stress treatment include mitochondrial therapy, stem cell transplantation, inhibitors and scavengers of free radicals, excitatory amino acid antagonists, hyperbaric oxygen (HBO) therapy and hypothermic treatments [18]. Compounds such as metformin [19], coenzyme Q10 [20] and mitoquinone [21], which directly regulate mitochondrial functions, seem to be promising. Experimental treatment of animals subjected to HI with selected antioxidants also results in a decrease in brain damage and ROS production [22,23]. It has also been shown that HBO treatment significantly reduces ROS production and brain damage in a hypoxic-ischemic rat model of birth asphyxia [24]. However, the exact effects of these treatments on HI injury and their ability to attenuate oxidative stress is still not fully known. To date, the only commonly accepted clinical treatment for birth asphyxia cases is therapeutic hypothermia. Therapeutic hypothermia started within 6 h after HI can reduce the risk of death or brain damage [25]. However, the above-mentioned treatments are either associated with adverse effects or are not very effective [26,27,28]; therefore, identifying new effective therapies is still a subject of investigations.

The role of metabotropic glutamate receptors (mGluR) subtypes in the pathophysiology of human disorders such as schizophrenia, depression, drug addiction, anxiety, Alzheimer’s and Parkinson’s diseases, and ischemic brain damage have been the subject of attention for several years [29,30,31,32]. Recently, group II metabotropic glutamate receptors and their role in HI have drawn the attention of scientists. mGluR2 and mGluR3 are negatively coupled to cyclic adenosine monophosphate (AMP) formation and act as presynaptic autoreceptors that regulate glutamate transmission. Their localization at the periphery of the synaptic cleft enables a response to any excessive glutamate release that leads to the escape of the neurotransmitter from the synaptic active zone; the activation of mGluR2/3 suppresses this release [33,34]. The inhibitory effects induced by mGlu2/3 receptor activation are mediated by a reduction in intracellular cyclic AMP levels or by decreased activity of voltage-gated Ca^2+^ channels [35]. mGluR3 is also localized on glial cells, and it has been shown that mGluR3 activation stimulates the release of neuroprotective transforming growth factor β (TGF-β) [36,37].

Because of their actions, mGluR2/3 appear to be promising targets for reducing excitotoxicity and neuronal injury. Preclinical studies have demonstrated that the activation of mGluR2 and/or mGluR3 by selective agonists results in neuroprotection in experimental models of ischemia in adult and developing animals [38,39,40,41]. *N*-acetylaspartylglutamate (NAAG) is the most abundant and frequently occurring peptide transmitter in the mammalian nervous system. It is also a full agonist of mGluR3. NAAG activates the mGluR3 at presynaptic sites, inhibiting the release of neurotransmitters, and acts on mGluR3 on glial cells, stimulating the release of neuroprotective growth factors from these cells [42]. Probably NAAG can also enhance glutamate uptake, as it was shown that a highly potent group II mGluR agonist DCG-IV induce glutamate uptake in astrocytes [42,43]. NAAG has been shown to be protective in focal cerebral ischemia and a neonatal rat model of hypoxia-ischemia. The application of NAAG in a short time before or after hypoxia-ischemia protects neurons from excessive NMDA receptor stimulation and reduces brain damage and pro-apoptotic caspase 3 expression [39,44]. We showed in our previous study that the application of NAAG up to 6 h after HI decreases oxidative stress, most likely by reducing excitotoxicity, which may explain its neuroprotective effects [38]. Based on the papers cited above, most studies have mainly investigated the effect of NAAG application after ischemic insult. Data on the neuroprotective effect of NAAG application a short time before HI are very limited [39,45], and there are not enough data to assert that the neuroprotective mechanism is similar to that observed for NAAG post-treatment. Because extracellular NAAG is quickly metabolized by the astrocytic enzyme glutamate carboxypeptidase II (GCP-II) [46,47,48], it would be interesting to investigate if the NAAG administered even earlier before HI still results in the neuroprotection and to identify the time window for the neuroprotective effect of NAAG pretreatment. It would also be interesting to determine if NAAG pretreatment results in attenuation of HI-evoked oxidative stress, as it has been observed when NAAG is applied after HI, the effect probably mediated by the inhibition of excitotoxicity by the blockade of glutamate release from presynaptic endings, resulting from the activation of presynaptic mGluR3 by NAAG [38].

In the present investigations, we evaluated the effect of NAAG applied at 24 or 1 h before experimental birth asphyxia in 7-day old rats and determined the result of this pretreatment on oxidative stress markers. We tried also to determine which antioxidant mechanisms are involved in this process.

## 2. Material and Methods

### 2.1. Ethics Approval and Consent for Participation

All experiments described in this paper were approved by the 4th Local Ethical Committee (263/2017) based in Warsaw, Poland and were performed following Polish governmental regulations (Dz.U.97.111.724), the European Community Council Directive of 24 November 1986 (86/609/EEC) and with Directive 2010/63/EU. All surgeries were performed under isoflurane anesthesia and all efforts were made to minimize animal suffering and the number of animals used.

### 2.2. Experimental Hypoxia-Ischemia

Neonatal hypoxia-ischemia was induced in rats on postnatal day 7 (P7) according to Rice et al. [49]. Wistar rat pups of both sexes were anesthetized (4% for induction, and 1.5–2.0% for maintenance, in 0.6:1 nitrous oxide and oxygen). The surgery involved unilateral interruption of blood flow to the brain by cutting off the left common carotid artery between double ligatures of silk sutures, followed by 75 min of hypoxia (7.5% oxygen in nitrogen) in a humidified chamber at 35 °C. The opposite artery remained undamaged and was therefore used as an internal control. After hypoxic treatment, the rats were returned to their mothers and were maintained in climate-controlled rooms (21 to 24 °C; 50% to 55% humidity) with diurnal lighting (12:12-h light/dark photoperiod). Sham-operated rats were used as controls. The condition of the animals, which remained in the experiment for fourteen days, was evaluated twice per day and, if necessary, an anesthetic was applied locally.

### 2.3. Drug Application

The selective mGluR3 agonist *N*-acetylaspartylglutamate (NAAG) (5 mg/kg of body weight), group II mGluR selective antagonist LY341495 (1mg/kg of body weight) and NMDA receptors antagonist memantine (5 mg/kg of body weight) were administered intraperitoneally at 24 or 1 h before HI (all chemicals Tocris Bioscience, Bristol, UK). The doses of the agonist and antagonists were determined based on our previous experiments and the literature [24,38,39]. Sham-operated and HI control rats were injected with saline.

### 2.4. Evaluation of Brain Damage

Brain damage was reflected by a deficit in the wet weight of the ipsilateral (left) ischemic hemisphere and is expressed as a percentage of the wet weight of the contralateral (right) control hemisphere. Two weeks after HI (PND 21), pups were anesthetized with a lethal dose of phenobarbital and decapitated. The brains were isolated, and the hemispheres were separated along the longitudinal fissure of the brain and weighed to the nearest 0.1 mg.

Histological evaluation of brain damage was performed on brains isolated seven days after HI. Animals were anesthetized and then transcardially perfused with phosphate-buffered saline (PBS) followed by a fixative solution (4% paraformaldehyde in PBS, pH 7.4). The brains were removed and post-fixed for 3 h at 4 °C in the same fixative solution. Following post-fixation, the brains were cryoprotected overnight in 30% sucrose solution, frozen on dry ice and stored at −70 °C. The brains were cut into 20–30 μm coronal sections on a cryostat. The sections were stained with 0.5% Cresyl violet according to a Nissl staining protocol for the histological assessment of neuronal cell damage. The number of living cells was counted in the cortex in the visual field under 200× magnification (250 μm × 250 μm) and in a CA1 area of 100 μm in length using the AxioVision imaging program (Carl Zeiss, Aalen, Germany).

### 2.5. Tissue Preparation for Biochemical Analysis

Brain tissues were collected for biochemical analyses 3 h after HI. The rats were decapitated, and the brain tissue samples containing the cerebral cortex and hippocampus were taken from both hemispheres for further examination. Tissues from the ipsilateral and contralateral hemispheres were homogenized separately in buffers prescriptive for each analysis. The protein concentration of the homogenates was determined by the Bradford method and the homogenates were used for further analyses.

### 2.6. Determination of ROS Level

The level of ROS in the cerebral hemispheres was determined with 2,7-dichlorofluorescein acetate (DCF-DA), which is de-esterified to dihydrodichlorofluorescein (H2DCF) when it comes in contact with cytoplasmatic esterases and is then oxidized by ROS to dichlorofluorescein (DCF), which is strongly fluorescent. Brain homogenates were placed in 40 mM Tris-HCL buffer (pH.7.4) and incubated with 25 μM DCF-DA in a 96-well plate for 30 min at 37 °C. The DCF fluorescence was then read with a multifunctional microplate reader (FLUOstar Omega, BMG LABTECH, Ortenberg, Germany) at 488 nm excitation and 530 nm emission wavelength. The relative fluorescence units (RFUs) of the homogenates were calculated per 1 mg of protein.

### 2.7. Determination of Glutathione Concentration

Brain tissue homogenates in 25 mM HEPES buffer (pH 7.4) containing 250 mM sucrose were centrifuged at 1000× *g* for 5 min at 4 °C. The supernatants were collected to measure the glutathione concentration using the Glutathione Assay Kit, Fluorimetric (Sigma-Aldrich, city, Saint Louis, MO, USA) according to the instructions provided by the manufacturer.

### 2.8. Determination of Antioxidant Enzyme Activity

#### 2.8.1. Superoxide Dismutase

Brain tissue homogenates suspended in 20 mM HEPES buffer (pH 7.2), containing 1 mM EGTA, 210 mM mannitol and 70 mM sucrose per 1 g of tissue were centrifuged at 1500× *g* for 5 min at 4 °C. The supernatant was collected to determine SOD activity using the Superoxide Dismutase Assay Kit (Cayman Chemical, Ann Arbor, MI, USA) according to the instructions provided by the manufacturer. The activity of the enzyme is expressed as the number of enzymatic units per milligram of protein (U/mg protein).

#### 2.8.2. Glutathione Peroxidase (GPx)

Brain tissue homogenates suspended in 50 mM Tris-HCl buffer (pH 7.5) containing 5 mM EDTA and 1 mM Dithiothreitol (DTT) per 1 g of tissue were centrifuged at 10,000× *g* for 15 min at 4 °C. The supernatants were collected to determine GPx activity using the Glutathione Peroxidase Assay Kit (Cayman Chemical, Ann Arbor, MI, USA) according to the instructions provided by the manufacturer.

#### 2.8.3. Catalase

Homogenates suspended in 50 mM potassium orthophosphate buffer (pH 7.0) containing 1 mM EDTA were centrifuged at 10,000× *g* for 15 min at 4 °C. The supernatants were collected for enzyme activity determination using the Catalase Assay Kit (Cayman Chemical, Ann Arbor, MI, USA) according to the instructions provided by the manufacturer.

### 2.9. Determination of TGF-β Concentration

Brain tissue homogenates suspended in PBS were centrifuged at 5000× *g* for 5 min and assayed using a Rat TGF-β ELISA Kit (MyBioSource, Inc., San Diego, CA, USA) according to the user manual.

### 2.10. Statistical Analysis

The results are expressed as the means ± SEMs of each experimental group. Statistical analysis was performed using a one-way ANOVA test with Dunnett’s post hoc test for significant differences between groups (GraphPad Prism 5; GraphPad Software Inc., La Jolla, CA, USA). The differences were considered statistically significant when *p* value was less than 0.05.

## 3. Results

### 3.1. The Effect of NAAG Application on HI-Induced Brain Damage

NAAG injected 24 or 1 h before HI significantly attenuated the weight deficit of the ischemic hemisphere, as examined 14 days after HI, and resulted in less damage to the ipsilateral (ischemic) hemisphere, as examined 7 days after insult. This indicates that the mGluR3 agonist had a neuroprotective effect.

**Figure 1:**
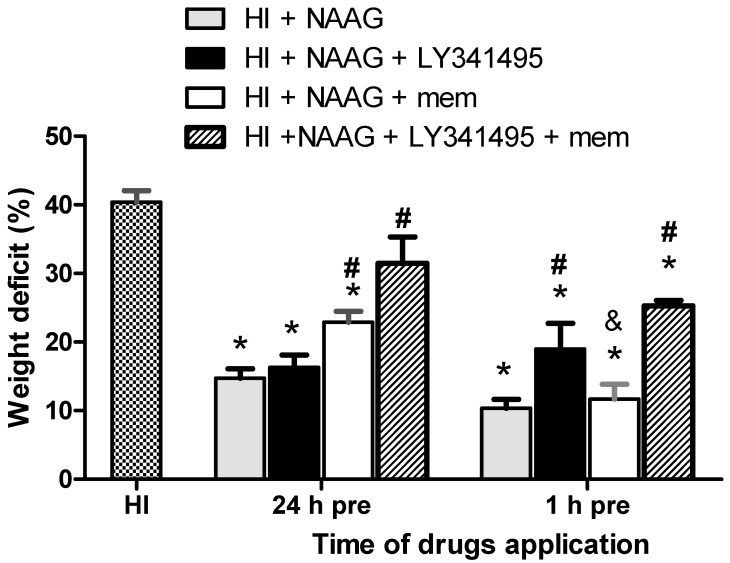
Effect of the application of group II mGluR antagonist LY341495 and NMDA receptor antagonist memantine (applied alone and in combination) on NAAG effect on the weight deficit of the ipsilateral hemisphere after HI. All drugs were applied i.p. 24 or 1 h before HI insult. The weight deficit is expressed as the percentage of the weight of the contralateral (right) hemisphere. The results are presented as the means ± SEM, *n* = 4–9; * *p* < 0.001 compared to the HI group; # *p* > 0.01 compared to the HI + NAAG group; & *p* > 0.005 comparison between NAAG + memantine applied at two different times before HI.

The 40% weight deficit of the ipsilateral hemisphere observed two weeks after HI was significantly reduced to 15% (*p* < 0.001, F_1,10_ = 36.7) and 10% (*p* < 0.001, F_1,16_ = 107) after i.p. NAAG injections 24 or 1 h before HI, respectively (Figure 1). The results show that the application of NAAG 1 h before HI resulted in more effective neuroprotection than application 24 h before HI. This difference was not big but statistically significant (*p* < 0.05, F_1,13_ = 4.86).

The application of NAAG to sham-operated animals did not produce any changes in the weight of the brain hemispheres, and HI did not result in the changes in the weight of the contralateral hemisphere (data not shown).

LY341495 applied together with NAAG 24 h before HI did not change the protective effect of NAAG, whereas applied with NAAG 1 h before HI significantly increased the weight deficit of the ipsilateral hemisphere (19% compared to 10% observed for NAAG applied 1 h before HI; *p* > 0.01, F_1,10_ = 8). However, we did not observe statistically significant differences in brain weight deficits between groups applied with a mixture of NAAG and LY341495 24 and 1 h before HI. Memantine applied together with NAAG 24 h before HI significantly diminished protective effect of NAAG (weight deficit of ipsilateral hemisphere—23%; *p* > 0.005, F_1,7_ = 18.71 compared to NAAG group). The application of memantine together with NAAG 1 h before HI did not change the protective effect of NAAG; however, we did not observe the additive effect of these two compounds either.

The application of NAAG in a mixture with antagonists of group II mGluRs and NMDA receptors 24 h or 1 h before HI resulted in a significant increase in ipsilateral brain hemisphere weight deficit compared to NAAG application alone (34.5% and 25% for drugs applied 24 h or 1 h before HI, respectively; *p* > 0.01, F_1,4_ = 22.5 and *p* > 0.001, F_1,10_ = 38.84 compared to NAAG groups, respectively).

In our further experiments, we concentrated on the investigation of the effects of NAAG application 24 and 1 h before HI.

**Figure 2:**
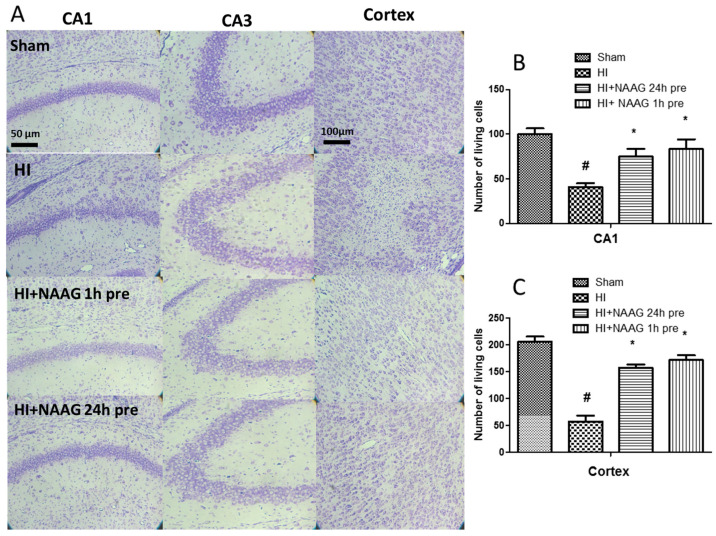
Neuroprotective effect of NAAG application observed in the CA1 and CA3 region of the hippocampus and the cerebral cortex of the ipsilateral hemisphere 7 days after HI (**A**). Quantification of surviving neurons in (**B**) the central part of the CA1 region (100 µm in length) and (**C**) cortex (250 μm × 250 μm area). NAAG was applied i.p. 24 h or 1 h before HI. The microphotographs show the ipsilateral hemisphere. # *p* < 0.005 compared to sham operated group; * *p* < 0.05 compared to HI group.

Cresyl violet staining showed that HI resulted in significant neuronal loss and disorganization of neurons in the CA1 region of the hippocampus, and neuronal damage in the cortex of the ipsilateral hemisphere. The number of living neurons observed in the central part of the CA1 region was reduced to 41% of that in the control (from 100 ± 9 to 41 ± 6 neurons, *p* < 0.005, F_1,4_ = 59.33) (Figure 2B) and to 27% in the cortex (from 206 ± 12 to 57 ± 14 neurons, *p* < 0.005, F_1,4_ = 114.39) (Figure 2C). The application of NAAG 24 or 1 h before HI significantly reduced the number of neurons lost in both brain regions. The number of surviving neurons in the analyzed area of the CA1 region increased significantly to 75% and 83% after NAAG injections at 24 h before HI and after NAAG injection 1 h before HI, respectively (*p* < 0.05, F_1,5_ + 13.2 and *p* < 0.05, F_1,5_ = 13.81, compared to HI, respectively).

The NAAG application also resulted in a significant increase in the number of living neurons observed in the cortex, with the number reaching 75% and 83% of that in the control group after NAAG application given 24 and 1 h before HI, respectively (*p* < 0.005, F_1,5_ = 68.55 and *p* < 0.005, F_1,5_ = 70.23 compared to HI, respectively). There was no significant difference between the group injected 24 h before and the group injected 1 h before HI. No changes evoked by HI or NAAG treatment were observed in the hippocampal CA3 region.

### 3.2. The Effect of NAAG Application on Changes in ROS Level in Rat Brain after HI

**Figure 3:**
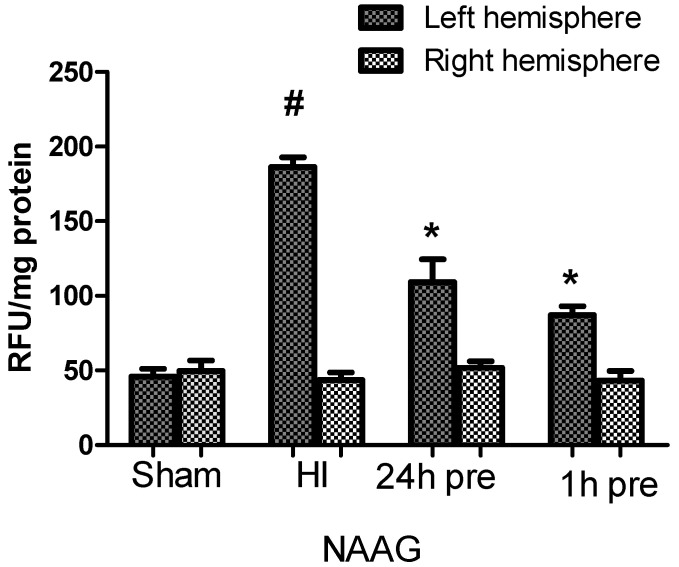
Effects of NAAG application on changes in ROS levels observed in the rat brain after HI. The results are presented as the means ± SEM, *n* = 6; statistically significant differences: * *p* < 0.001, compared to the HI group; # *p* < 0.001 compared to the sham operated group.

HI caused a 3-fold increase in the ROS level in the ipsilateral hemisphere compared to that in control animals. There were no changes in ROS concentration in the contralateral hemisphere (Figure 3).

I.p. injections of NAAG 24 or 1h before HI resulted in reductions in the increase in ROS by 41% and 54%, respectively, compared to the HI group (*p* < 0.001 for both groups, F_1,9_ = 18.98 and F_1,9_ = 128.4 for NAAG applied 24 and 1 h before HI, respectively). These results indicate that the NAAG application at either time before HI resulted in a reduction in HI evoked in ROS production; moreover, there was no statistically significant difference between the effect of NAAG application at the two time points.

HI did not result in changes in the ROS level in the contralateral (right) hemisphere, and the NAAG application did not affect the ROS level.

### 3.3. The Effect of NAAG Application on HI Induced Changes in SOD Activity

**Figure 4:**
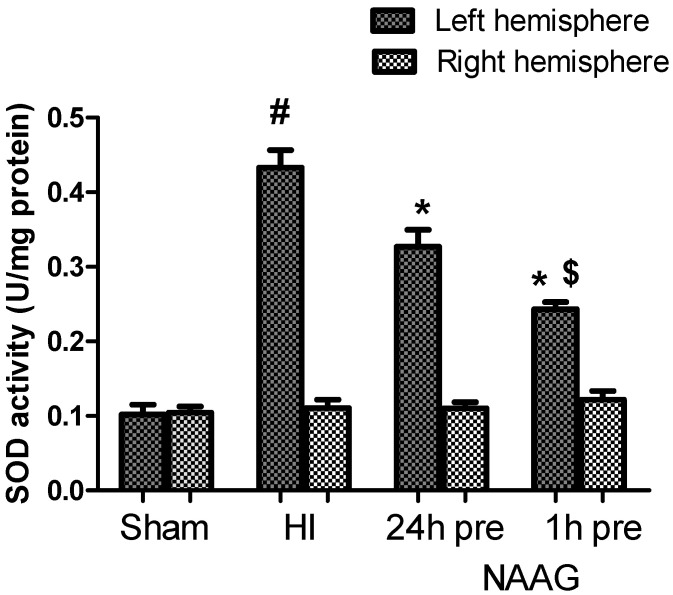
Effect of NAAG application on HI-induced changes in SOD activity. The results are presented as the means ±SEM, *n* = 6; statistically significant differences: * *p* < 0.01, compared to the HI group; # *p* < 0.001 compared to the sham operated group; $ *p* < 0.01 compared to the NAAG 24 h pre group.

Changes in the activity of superoxide dismutase (SOD) in both cerebral hemispheres were examined. The results show a 4-fold increase in SOD activity measured 3 h after HI in the ipsilateral hemisphere compared to the control group (Figure 4). In animals injected with NAAG 24 or 1h before HI, SOD activity in the ipsilateral hemisphere decreased significantly by 24% (*p* < 0.01, F_1,10_ = 10.62) and 44% (*p* < 0.001, F_1,10_ = 57.53), respectively, in comparison to that in the HI group. The observed decrease in activity SOD was significantly greater when NAAG was administered 1 h before HI (*p* < 0.01, F_1,10_ = 11.23). We did not observe changes in SOD activity in the contralateral hemisphere.

### 3.4. The Effect of NAAG Application on Changes in GPx Activity after HI

**Figure 5:**
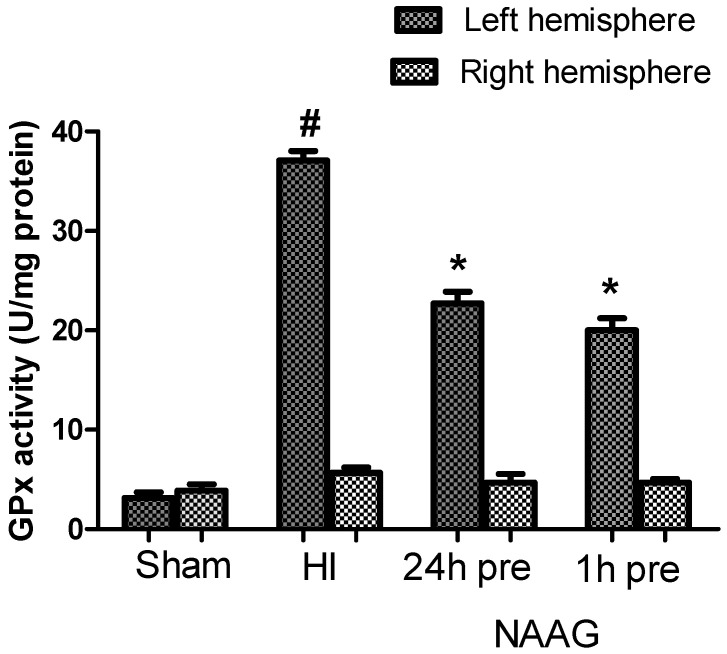
Effect of NAAG application on changes in glutathione peroxidase activity induced by HI. The results are presented as the means ± SEM, *n* = 6–7; statistically significant differences: * *p* < 0.001 compared to the HI group; # *p* < 0.001 compared to the sham operated group.

This study shows that the average GPx activity in both hemispheres of the brains of control rats was 3 U/mg protein on. GPx activity in the ipsilateral hemisphere after HI increased more than 10-fold and reached the value of 35 U/mg of protein (Figure 5).

The application of NAAG 24 h before HI and the application of NAAG 1 h before HI significantly decreased GPx activity in ipsilateral hemispheres by 39% (*p* < 0.001, F_1,11_ = 92.55) and 46%, (*p* < 0.001, F_1,11_ = 126.18), respectively. There was no significant difference between the groups injected with NAAG at the two different time points.

We also observed a slight increase in GPx activity induced by HI in the contralateral hemispheres (5.76 U/mg protein); however, this increase was not statistically significant.

### 3.5. The Effect of the NAAG Application on Changes in GSH Level after HI

**Figure 6:**
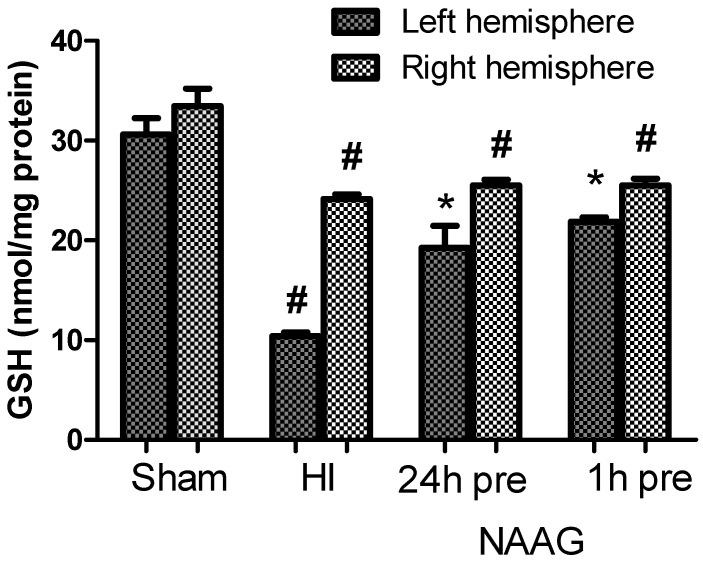
Effect of NAAG application on changes in the GSH concentration in the brains of rat pups after HI. The results are presented as the means ± SEM, *n* = 5–6; statistically significant differences: * *p* < 0.005 compared to the H-I group, # *p* < 0.001 compared to the sham operated group.

The GSH level measured in the brain homogenates of both hemispheres of control rat pups was 32 ± 1.5 nmol/mg protein in. HI caused a decrease in GSH content in both hemispheres: the GSH content in the ipsilateral hemisphere decreased to 34% of that in the control group (10.4 nmol/mg protein; *p* < 0.001, F_1,11_ = 125.44) and that in the contralateral hemisphere decreased to 72% of that in the control group (24.9 nmol/mg protein; *p* < 0.001, F_1,11_ = 22.91) (Figure 6).

The application of NAAG 24 or 1h before HI resulted in the significant restoration of GSH concentration in the ipsilateral hemisphere to 63% of that in the control group (*p* < 0.005, F_1,9_ = 18.88 compared to the HI group) and 71.4% of that in the control group (*p* < 0.001, F_1,10_ = 100.49 compared to the HI group), respectively. The differences in the GSH concentration between the groups injected with NAAG 24 h or 1 h were not statistically significant.

We did not observe changes in the GSH concentration in the contralateral hemisphere after the NAAG application compared to that in the HI group; it remained at approximately 25 nmol/mg of protein.

### 3.6. The Effect of NAAG Application on the Changes in Catalase Activity Observed after HI

**Figure 7:**
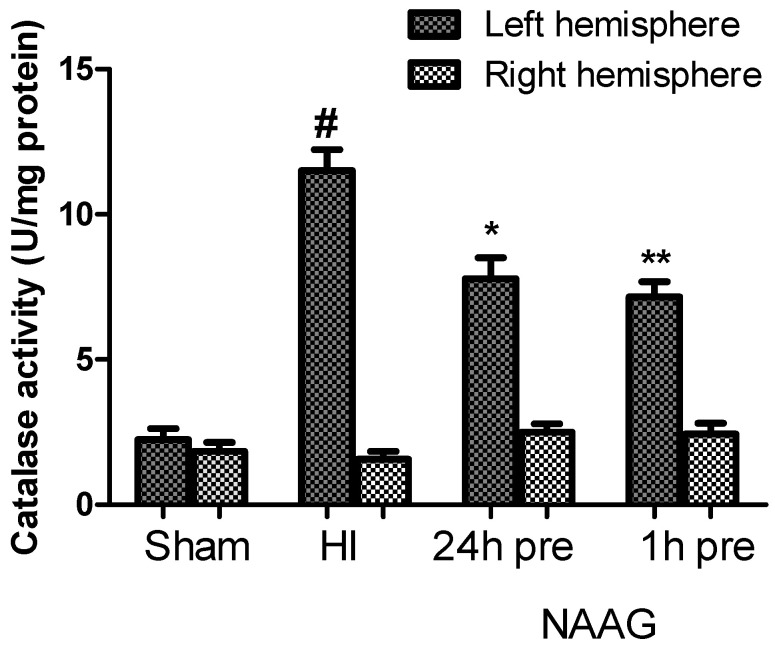
Effect of NAAG application on HI induced changes in catalase activity. The results are presented as the means ±SEM, *n* = 6; statistically significant differences: * *p* < 0.005, ** *p* < 0.001 compared to the HI group; # *p* < 0.001 compared to the sham operated group.

The average activity of catalase measured in the brains of sham operated rats (both hemispheres) was 2 U/mg protein. HI increased catalase activity in the ipsilateral hemisphere almost 6-fold compared to control values, with the catalase activity reaching 11.5 u/mg protein (*p* < 0.001, F_1,10_ = 129.48) (Figure 7).

The application of NAAG 24 or 1h before HI resulted in a significant decreases in catalase activity in the ipsilateral hemispheres to 67% (*p* < 0.005, F_1,10_ = 13.3) and 62% (*p* < 0.001, F_1,10_ = 23.71) of the activity measured in the HI group, respectively. There was no statistically significant difference between the groups that received NAAG at the two different time points.

We observed no changes in the catalase activity in the contralateral hemisphere after HI, or after the application of NAAG.

### 3.7. The Effect of NAAG Application on Changes in the TGF-β Concentration after HI

**Figure 8:**
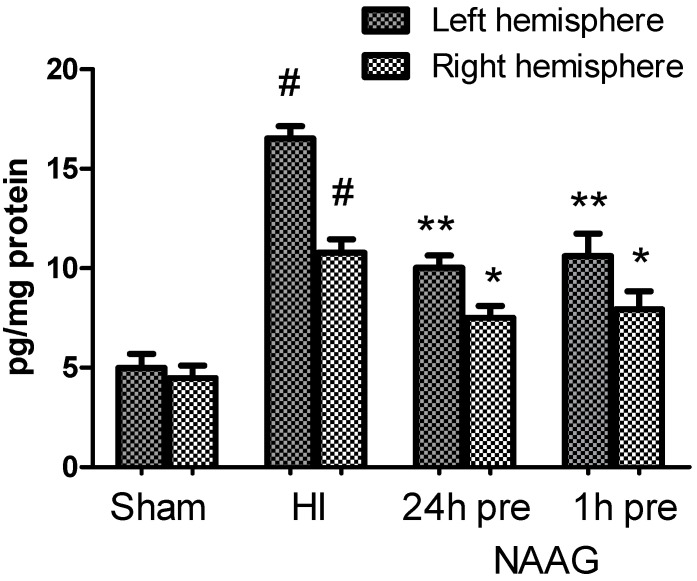
Effect of NAAG application on HI-induced changes in the TGF-β concentration. The results are presented as the means ±SEM, *n* = 6; statistically significant differences: * *p* < 0.05, ** *p* < 0.001 compared to the HI group; # *p* < 0.001 compared to the sham operated group.

The TGF-β concentration measured in the brains in the sham operated rats ranged from 3.5 to 4.0 pg/mg protein. HI significantly increased the TGF-β concentration to 17.2 pg/mg protein in the ipsilateral hemisphere and to 10.8 pg/mg protein in the contralateral hemisphere (*p* < 0.001, F_1,10_ = 151.8 and *p* < 0.001, F_1,10_ = 46 compared to the sham-operated group, respectively)(Figure 8). However, the increase observed in the contralateral hemisphere was significantly lower than that observed in the ipsilateral hemisphere (*p* < 0.001, F_1,10_ = 39.4). The application of NAAG 24 or 1 h before HI significantly decreased the TGF-β concentration to approximately 10 pg/mg protein in both groups (*p* < 0.001, F_1,10_ = 21.2 and *p* < 0.001, F_1,10_ = 55.5, respectively). The TGF-β concentration in the contralateral hemisphere was also significantly decreased to 7.7 ± 0.12 pg/mg protein by NAAG application at both tested time points (*p* < 0.05, F_1,10_ = 6.36 and *p* < 0.005, F_1,10_ = 12.96 for NAAG applied 24 h or 1 h before HI, respectively). However, the TGF-β concentration measured after the NAAG application at both tested time points was still significantly higher than that in both brain hemispheres of the sham operated group.

## 4. Discussion

Since its discovery in the mid-1960s, NAAG has been recognized as the most abundant peptide neurotransmitter in the mammalian central nervous system [50]. The important role of NAAG in neuroprotection has been demonstrated by many scientists; however, the exact mechanism of the observed neuroprotection is still not well understood [47,51].

The results presented in this paper show the neuroprotective effects of NAAG applied before HI in a neonatal rat model of birth asphyxia. NAAG applied 1 or 24 h before HI significantly reduced brain weight deficits. Application of antagonist of mGluR2/3, LY341495 24 h before HI did not affect NAAG neuroprotection, whereas the application of memantine significantly decreased it. A significant decrease of NAAG neuroprotection was observed when LY341495 was applied together with NAAG 1 h before HI but memantine did not change this effect. It was shown before that memantine applied 24 h before ischemic insult did not prevent brain damage induced by HI in 7-days old rats or global cerebral ischemia in gerbils [52]. Presented results suggest that pretreatment with NAAG 24 h before HI may induce mild excitotoxicity resulted from NMDA receptors activation that triggers the defense system in neurons. However, the mechanism of neuroprotection resulted from the application of NAAG 1 h before HI seems to be slightly different and involving the activation of mGluR3 receptors. Interestingly, the application of a mixture of antagonists together with NAAG resulted in a significant reduction of NAAG’s neuroprotective effect, which is difficult to explain and needs additional investigation.

Further experiments, concentrated on the neuroprotective effect of NAAG showed that the application of NAAG increased the number of living cells in the CA1 region of the hippocampus and cortex of the ipsilateral hemisphere. These results are in agreement with the results presented by Cai et al. [39] and Hemelrijck et al. [44], although their observations of the effects of pretreatment with NAAG were limited to applications no earlier than 1 h before ischemia or hypoxia-ischemia. Our results show that NAAG pretreatment may result in the protection of neurons even when administered 24 h before HI.

Newborns, especially preterm newborns, are particularly sensitive to ROS, as the immature brain, especially in neuronal membranes, is rich in polyunsaturated fatty acids and in redox-active iron, which may be an additional source of oxygen radicals. Under ischemic conditions the antioxidant defense system fails to protect neurons from oxidative stress due both the overproduction of ROS and the effeteness of defense-related forms of antioxidant enzymes; therefore the restoration of the antioxidant system is very important. Many reports have confirmed that HI results in an increase in activity of key antioxidant enzymes, but decreases the GSH concentration [24,38,53,54]. It has been shown that the activation of mGluR3 reduces glucose-induced oxidative injury in neuronal cell cultures by increasing the GSH concentration and that the application of NAAG a short time before or after ischemia protects against oxidative stress and apoptosis in rat models of ischemia [38,44,55]. However, no data is showing how the pretreatment with NAAG affects changes in antioxidant enzymes activity after HI. The results of the experiments presented in this paper show for the first time, that the application of NAAG before HI prevents oxidative stress, resulting in significant decreases in ROS levels and the partial restoration of the GSH concentration; however, we did not observe additional activation of antioxidant enzymes, and the activities of SOD, catalase and GPx were significantly lower than those observed in HI group. Moreover, the effect of the NAAG application did not differ between experimental groups, indicating that the neuroprotective mechanism can be triggered and remain active even for 24 h. This, together with the fact that NAAG reduced HI-evoked cell death, may suggest that pretreatment with NAAG does not result in increased effectiveness of the antioxidant defense system but rather reduces ROS production.

In our earlier publication, we postulated that the neuroprotective effect of NAAG applied after HI results from the activation of presynaptic mGluR3, which inhibits glutamate release [38]. However, the neuroprotection observed in this study is probably not an effect of the inhibition of glutamate release. An increased level of NAAG in the extracellular space increases the activity of astrocytic glutamate carboxypeptidase II (GCP II), which degrades NAAG into glutamate and N-acetyl-aspartate (NAA) [47]. GCP II is present in the immature brain, although whether its activity is essential for normal embryonic development is disputable [56,57,58]. It has also been shown that conditions that lead to excess synaptic activity (ischemia and inflammation) enhance GPC II activity, resulting in an increase in the glutamate level [59,60].

In the present experiments, NAAG was applied 1 or 24 h before HI, and, likely, both NAAG and glutamate generated by its metabolism were already neutralized when the animals were subjected to HI conditions.

Reports from in vivo and in vitro studies have shown that an increase in the level of TGF-β can rescue neurons from ischemia-induced excitotoxicity and inflammation; TGF-β also promotes anti-apoptotic actions and neuroregeneration [61]. It has been shown that the neuroprotection observed after NAAG applications results mainly from the activation of glial mGluR3 and the induction of increased production TGF-β in the brain [36]. The inhibition of GCPII has been demonstrated to reduce neurodegeneration in rats after transient middle carotid artery occlusion [62] and in neuronal cell cultures [37,63]. It has been postulated that neuroprotection resulted from GPCII inhibition requires TGF-β and is partially mediated by the promotion of its release [37]. However in the same in vivo model of cerebral ischemia, increasing TGF-β concentration did not alter the ischemic injury [37]. Moreover, recently it was suggested that oxidative stress and apoptosis resulted from ischemia in immature and adult rats can be alleviated via inhibition of TGF-β signaling [64,65].

Our investigations showed that HI evoked a significant increase in the TGF-β concentration in both hemispheres, which is in agreement with reports by other authors [61,66]; however, we did not observe any additional increase in the TGF-β concentration in HI groups pretreated with NAAG. The results show that TGF-β concentration, although still higher than that in the control group, was significantly decreased. Therefore, we exclude this process as a part of the mechanism of neuroprotection elicited by NAAG pretreatment.

The application of NAAG under physiological conditions, as it takes place in our experiments, in addition to activating mGluR3, increases also the activity of synaptic NMDA receptors containing GluN2A subunits and inhibits extrasynaptic receptors containing GluN2B [67]. It has been shown that the activation of neuronal NMDA receptors by NAAG can result in a calcium current comparable to the current induced by NMDA [68] and that Ca^2+^ signaling resulting from the activation of synaptic NMDA receptors can trigger the antioxidative defense system, probably through the activation of the thioredoxin system [69]. However, the reaction catalyzed by GCPII liberates glutamate, which subsequently activates extrasynaptic glutamate receptors present on surrounding neurons and astrocytes [59]. The fact that LY341495 did not inhibit neuroprotection resulted from NAAG application 24 h before HI, whereas memantine significantly reduced this effect suggests the involvement of NMDA receptors in observed phenomenon. Excessive activation of glutamate receptors resulting from the NAAG application may create the conditions of mild excitotoxicity and trigger the defense mechanisms in neurons. We believe that this kind of effect of the NAAG application well in advance of HI may be considered as preconditioning. This phenomenon is induced by a variety of treatments that make neurons more resistant to a subsequent ischemic or hypoxic insult. The most commonly used treatment is the preconditioning with mild ischemia, although the neuroprotective effects of hypoxic, pharmacological, and hyperbaric oxygen preconditioning have also been demonstrated [70,71,72,73]. It has not been fully revealed how the preconditioned neurons adapt to subsequent ischemic insult, but many scientists agree that it involves multiple protective mechanisms.

## 5. Conclusions

The present results show that the NAAG pretreatment, applied both 24 h or 1 h before HI, results in a decrease in neuronal death and a significant reduction in ROS production. While the neuroprotective effect of NAAG applied 1 h before HI seems to be the combination of activation of mGluR3 and NMDA receptors, the neuroprotective effect of NAAG applied 24 h before HI is probably mainly connected with NMDA receptors.

Observed neuroprotection and ROS reduction may be explained by the induction of mild excitotoxicity that does not result in neuronal death but triggers the antioxidative defense system in neurons. This conclusion is additionally supported by the fact that NAAG pretreatment performed 24 h before HI results in a decrease in antioxidant enzymes activity and the TGF-β concentration, which excludes the involvement of the activation of astrocytic mGluR3. Moreover, this response to NAAG pretreatment is consistent with the commonly accepted mechanism of preconditioning. However, this theory requires further investigations.
